# Frequency Spectra Analysis of Drawbar Pulls Generated by Special Driving Wheels Improving Tractive Performance

**DOI:** 10.3390/s21092903

**Published:** 2021-04-21

**Authors:** Rudolf Abrahám, Radoslav Majdan, Katarína Kollárová, Zdenko Tkáč, Martin Olejár, Eva Matejková, Ľubomír Kubík

**Affiliations:** 1 Department of Transport and Handling, Faculty of Engineering, Slovak University of Agriculture in Nitra, Tr. A. Hlinku 2, 949 76 Nitra, Slovakia; rudolf.abraham@uniag.sk (R.A.); radoslav.majdan@uniag.sk (R.M.); zdenko.tkac@uniag.sk (Z.T.); 2 Information and Coordination Centre of Research, Faculty of Engineering, Slovak University of Agriculture in Nitra, Tr. A. Hlinku 2, 949 76 Nitra, Slovakia; 3 Department of Electrical Engineering, Automation and Informatics, Faculty of Engineering, Slovak University of Agriculture in Nitra, Tr. A. Hlinku 2, 949 76 Nitra, Slovakia; martin.olejar@uniag.sk; 4 Department of Statistics and Operations Research, Faculty of Economics and Management, Slovak University of Agriculture in Nitra, Tr. A. Hlinku 2, 949 76 Nitra, Slovakia; eva.matejkova@uniag.sk; 5 Department of Physics, Slovak University of Agriculture in Nitra, Tr. A. Hlinku 2, 949 76 Nitra, Slovakia; lubomir.kubik@uniag.sk

**Keywords:** fast Fourier transform, frequency component, lug wheels, tires, drawbar pull

## Abstract

Driving wheel operation is characterized by force interactions with the ground, manifested in the form of vibrations. Signals generated by driving wheels can be analyzed in the frequency spectrum of tractor drawbar pull. The paper presents the analysis of a drawbar pull signal generated by a tractor equipped with two types of special driving wheels and standard tires. Beside the evaluation of special driving wheels’ properties according to drawbar power, the frequency spectra of measured signals were analyzed using a fast Fourier transformation. The model spectrum intervals for the standard tires, spike tires, and blade wheels were calculated according to the number of rubber lugs, blades, or spikes and compared with the experimental results. The results showed that the specific frequencies typical for blades and spikes were identified in model spectrum intervals. In the case of standard tires, the spectrum components typical for rubber lugs of the tire tread pattern were not identified. The highest amplitude of the typical frequency component was detected in the case of blades wheels, which showed the highest difference in drawbar power in comparison with the standard tires. Smaller dimensions of spikes resulted in lower amplitude and lower difference in drawbar power in comparison with the standard tires.

## 1. Introduction

Vibrations are a typical demonstration of functionality for many machines. The signal analysis of machine vibrations is often used for diagnostics of technical conditions of various machines or identification of their individual parts’ operation [1]. Fast Fourier transformation (FFT) is widely used for signal analysis for many applications in engineering, science, mathematics, etc. Several mechanical features of rotary mechanisms change with speed [2,3]. The FFT analysis of the frequency spectrum helps to identify the characteristic frequency components because no obvious peaks appear in the spectrum. This method reduces the measured signals to characteristic components and associates the components with mechanical parts of the machine. The information about single mechanical parts and the whole machine can be obtained. The methods of measuring the vibration parameters of the drawbar pull signal present the great robustness of the method algorithm. The uncertainty of measurements is an essential part of the validation process. The quantification of uncertainty in prediction is an intractable problem using traditional models [4].

**Figure 1:**
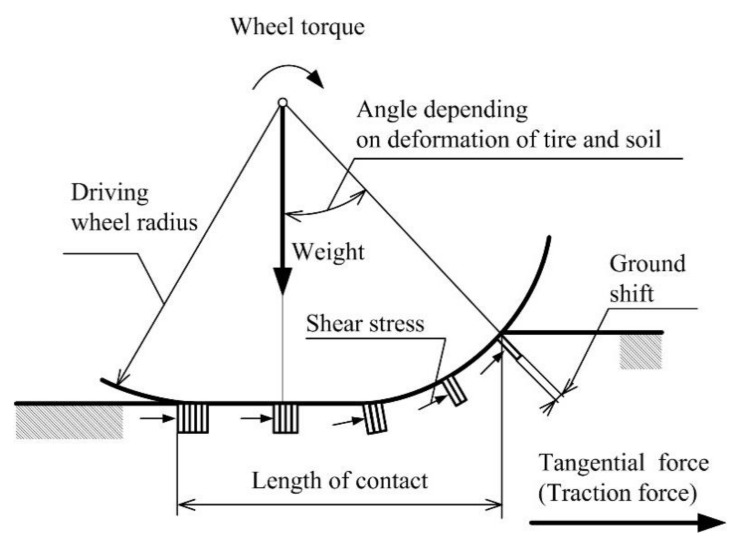
The elastic tire of a driving wheel on soil.

Driving wheels are parts of the machine generating vibrations due to forces’ interactions between the wheel and ground. Makharoblidze et al. [5] present that as a driving wheel moves, its lugs shift and cut the ground in the direction opposite to their movement (Figure 1). During the interaction of a driving wheel with the soil, there are traction forces between the support surface of the tire and soil, in particular the forces arising at cutting the ground brick with the side edges of the lugs. The support of the lugs in the ground, and the shift and cutting of bricks constrained between them yes, changes retain the meaning are possible only if the traction forces are thoroughly used, i.e., in the case of a wheel slip. The maximum shift is commensurable to the number of lugs in the gearing of the support surface with the ground. The maximum value of the ground shift is the product of the wheel slip and length of contact between the wheel and ground. Watyotha and Salokhe [6], Watyotha et al. [7], and Soekarno et al. years are not necessary [8] presented that lifting force acts in the opposite direction to weight in the case of the lugged wheel. The results of the authors mentioned above showed the relationship between the various lugs and lifting forces, which contributes to the driving wheel vibrations.

The frequency spectrum is typical for the design of wheels and the ground type. The amplitude and specific frequency of vibrations generated by the driving wheels affect the quality of the machine’s operation. Improvement of the driving wheels design of wheeled robots is aimed at reduction of vibrations negatively affecting the robot stability [9,10]. Vibrations are an undesirable phenomenon also typical for operation of road vehicles [11]. The main tire parameters influencing the wheel performance are tire diameter, tire width, dual tire, tire shape, lug shape, lug height, lug angle, lug spacing, tread material, inflation pressure, tire deflection rim width, and carcass construction [12]. One of the many factors affecting the wheel oscillation is the tire tread pattern type, mainly characterized by the elasticity, shape, and dimensions of the rubber lugs. A large space between neighboring rubber segments is typical for the tires of off-road vehicles and tractors because this parameter improves the tractive performance under off-road conditions. A similar principle uses the steel or rubber tracks [13,14,15] equipped with a higher number of grousers, better interacting with the ground in comparison with tires [16,17].

There are a lot of technical solutions proposed to improve the tire tractive performance based on high rubber lugs or additional steel segments of various shapes and geometry [18,19,20]. Considering the research results of the authors mentioned above, an original design of two types of special driving wheels was proposed. Two concepts used either the spikes in a tire tread pattern or steel blades near the driving wheel to interact with the ground and modify the properties of driving wheels with the standard tires. The main novelty of the presented study is an analysis of drawbar pull signals generated by the special driving wheels and standard tires to identify the characteristic frequency spectra, resulting in the driving wheel design. The influence of the driving wheels on vibrations and the machine stability was confirmed by several authors [9,10,11] and analyzed by various techniques. The authors measured signals by an accelerometer, and motors using their original Hall effect encoders yes, the change retains the meaning or rotation speed sensor. In addition to the evaluation of the special driving wheels’ tractive properties, the operation of various driving wheel types was analyzed from the signals of drawbar pull using the fast Fourier transformation [1,21,22,23]. The signals were obtained from the load cell connected between the test and load tractor. 

As mentioned above, the special driving wheels help improve the tractive performance of various vehicles. The paper presents the identification of vibration signals of the driving wheels using the frequency spectra analysis. The drawbar pull signals from the strain gauge load cell were used for evaluation of the tractive properties of the special driving wheels. The experimental tests were performed under real operation conditions, with the experimental data recorded. In the first section, a design of the two types of the special driving wheels is described to characterize the main features affecting the components of the drawbar pull signals. In the second section, the drawbar power was calculated, and tractive properties of the special driving wheels and standard tires are presented. The robustness was expressed, and autocorrelations were estimated between the values of drawbar pulls. In the third section, the fast Fourier transformation was applied to analyze the drawbar pull signals, and model frequency intervals typical for the vibration signals due to soil–wheel interactions were calculated. The specific frequency components within model intervals were identified and their amplitudes compared.

## 2. Materials and Methods

### 2.1. Design of Driving Wheels

In this section, two special driving wheels, namely the spike tires and blade wheels, are presented. The principle and test of special driving wheels were published by [24,25,26]. Both were designed at the Slovak University of Agriculture in Nitra. The action of forces during the rotation of designed driving wheels is the same as the mechanics of lugged wheels.

**Figure 2:**
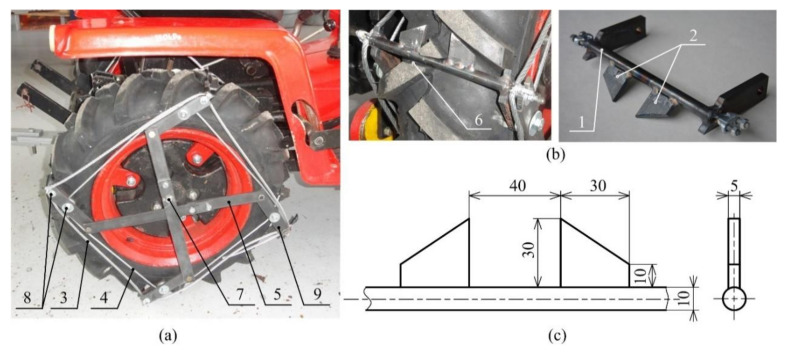
Spike tires: (**a**) spike tire on the tractor; (**b**) spike element; (**c**) dimensions of spikes: 1—support rod of the spike segment, 2—spike, 3—carrier wire strand, 4—control wire strand, 5—control mechanism, 6—groove in the tire, 7—safety screw, 8—pins, 9—spike element.

The design of the spike tires (Figure 2) was proposed with four spike elements (9), which consist of a support rod (1) and two spikes (2). Each element is placed in the tire groove (6) made in the tire tread pattern. The control (4) and carrier wire strands (3) connect all the spike elements together by pins (8). Removing a safety screw (7), the rods of the control mechanism (5) are released to allow an automatic protrusion (protruded position) of the spike elements due to wheel rotation. The control wire strand (4) helps to protrude all the spike elements and holds them in the same position. The carrier wire strand (3) holds all the spike elements in the tire trad-pattern during the wheel rotation. The spike elements are placed aback (base position) for road transportation. 

The spike elements were designed regarding the space in the tire tread pattern because they cannot exceed the tire diameter in the base position. The steel material S355 with good weldability was used for the spike segments and the control lever mechanism.

**Figure 3:**
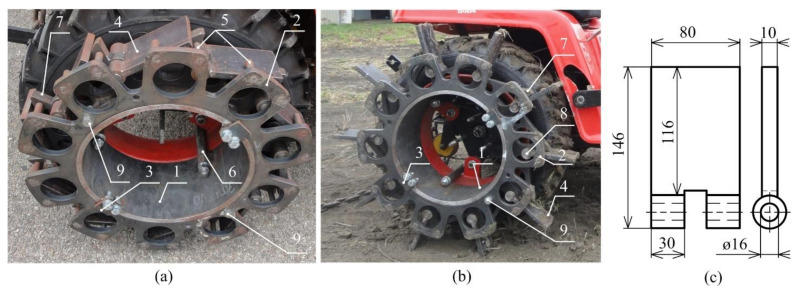
Blade wheels: (**a**) base position for road transportation; (**b**) protruded position for field conditions; (**c**) blade shape and dimensions: 1—support tube, 2—control disc, 3—stop bars, 4—blade, 5—pins, 6—fastening screw, 7—steel triangle, 8—pivot, 9—safety bolt.

A support tube (1) creates the body of the blade wheel (Figure 3). The support tube connects the blade wheel with the tractor driving wheel by means of three screws (6). Ten blades (4) are fulcrumed around the pivots (8), which are fastened to the support tube (1). A control disc (2) is also fulcrumed and secured from axial movement by means of two opposite stop bars (3). Removing one or two safety bolts (9) and turning the control disc (2), the pins (5) move the blades (4) to the protruded (Figure 3b) or base position (Figure 3a). Moving the tractor, the blades (4) can be automatically protruded, too. Removing the safety bolts (9) and moving the tractor back, the blades are automatically placed in the base position for road transportation. In the base position, the blades do not exceed the tractor wheel diameter. The steel triangles (7) support the protruded blades during the operation. The blade dimensions are shown in Figure 3c. The total length of the blade is 116 mm but only a 30 mm length extends over the tire. The space between the adjacent blades and the number yes, the change retains the meaning of blades limit the maximum working length.

Two special wheel types were proposed, using the spikes in the case of the spike tires (Figure 2) and blades in the case of the blade wheels (Figure 3). The blades are placed alongside the tractor wheel. The spikes are directly placed in the tire tread pattern of the tire. Both technical solutions do not affect the road transportation of the tractor using the tires, thereby improving the drawbar properties during the tractor operation on the soil or grass surface. This feature of both special driving wheels results in two lug or spike positions, namely the base position for road transportation and the protruded position for off-road operation.

### 2.2. Drawbar Pull Measurement System

**Figure 4:**
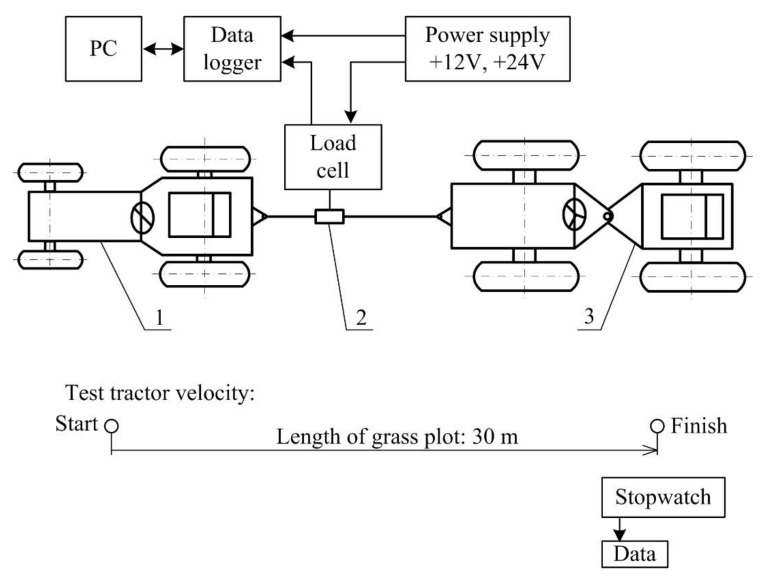
The system for measuring the drawbar pull of the tractor with different driving wheels: 1—test tractor; 2—load cell; 3—load tractor.

The measurement system shown in Figure 4 was used to analyze the frequency spectrum of drawbar pulls generated by different types of driving wheels. The test tractor was equipped with the standard tires with no device improving the tractive properties, the spike tires, and the blade wheels. The tires TS-02 6.5/75-14 4PR TT (Mitas, a. s., Prague, Czech Republic) were used in all the cases. Using the load tractor, the test tractor was loaded by two load levels. When the load tractor was in the neutral or third gear, the test tractor operated at a low or high load level. Providing the constant load, the test tractor operated at a steady state regime. The load cell was placed between the load and test tractor.

The main parts of the measurement system are as follows:

- The test tractor MT8-070 Mini (Agrozet, a. s., Prostějov, Czech Republic) with a gasoline engine with the volume capacity of 400 cm^3^, rated engine power of 8 kW, and rated engine speed of 3600 min^−1^ pulling the load tractor to generate the drawbar pull. Three types of driving wheels (the standard tires, the blade wheels, and the spike tires) were mounted to the test tractor. The curb weight of the test tractor was 310 kg. The driver weight was 90 kg. The weight distribution on axes of the test tractor was published by [27].
- The load tractor 4K-14 (Agrozet, a. s., Prostějov, Czech Republic) with a diesel engine with the volume capacity of 661.6 cm^3^, maximum output power of 13 kW loading the test tractor during the measurement of drawbar pull. The curb weight of the load tractor was 870 kg. The driver weight was 80 kg.
- The load cell EMS 150 (Emsyst, s. r. o., Trenčín, Slovak Republic) with a strain-gauge bridge in steel housing (with accuracy of 0.2% or ±20 N, rated capacity: 10 kN). Output signal (sensitivity of strain-gauge) was 2 mV/V. It means that the output signal in mV obtained at the nominal loading depends on the voltage supply of the strain-gauge in V. At the recommended supply 10 V and nominal loading 10 kN, the output signal was 20 mV. The uncertainty of the measurement was ±2 mV or ±20 N. The data logger HMG 3010 (Hydac GmbH, Sulzbach, Germany) is a high-performance portable measuring and data-logging device [28,29] with accuracy ≤0.1%. The sampling frequency was set to 1 kHz. Resolution was 12 bit. The sensitivity of the measurement range from 0 to 10 V was 10/4096, i.e., 2.4 mV/bit. The uncertainty of the measurement was ±2.4 mV. The total uncertainty of the measurement was ±4.4 mV or ±44 N.
- The power supply contains two accumulators (12 V) connected in series or parallel to supply the sensor and data logger with direct voltage (12 V or 24 V). The power supply was manufactured as a portable device.
- The measurement of drawbar pull was performed at the distance of 30 m. The time of one passage from the start to the end was measured by a stopwatch to determine the test tractor velocity, as shown in Figure 4. The tractor with each driving wheel type did not move in the driving tracts after previous measurements. The tests have never been repeated on the previous tracks. The grass plot was large enough to move the tractor into a new place for each measurement.
- A wheel gauge of the test and load tractor was 0.65 m and 0.85 m. Considering the increase in the wheel gauge of the test tractor with the blade wheel and tire width of both tractors, the load tractor moved on the grass plot surface, but was yes, the change retains the meaning partially disrupted by the special driving wheels. To eliminate the influence of the grass plot surface conditions on the breaking forces, the four-wheel drive load tractor with an adequate weight was used. The weight of the load tractor was more than two times higher than the test tractor.
- Soil properties affect the force interaction between the ground and wheels. The drawbar pull of the tractor with different types of driving wheels was measured at the same soil type of the grass plot and at the same soil moisture to eliminate the change of measured values due to soil properties. The total weight of the tractor is the main parameter that affects the drawbar pull. When the standard and spike tires were used, the rims of driving wheels were ballasted with the correct additional weight to reach the same tractor weight as in the case of the blade wheels. The drawbar pull measurements were performed in accordance with [30]. The test tractor was in the second gear (gear ratio *i_2_ =* 146.3) and operated at the rated rotation speed of the engine (3600 min^−1^).

To evaluate the drawbar properties of the special driving wheels, the drawbar pulls of the tractor with the standard tires, blade wheels, and spike tires were measured and statistically processed. The mean values of drawbar pulls and drawbar powers were calculated to represent the drawbar performance of the test tractor at two levels of load. One-way analysis of variance (ANOVA) was used to determine which means are significantly different from which others. The method used to discriminate among the means was Tukey’s significant difference procedure performed by the statistical software SAS EG (version 7.1).

### 2.3. FFT Analysis of Drawbar Pull Signals

Signals of measured drawbar pulls are represented in the time domain. However, they do not contain enough information for their comprehensive understanding. For that all, additional information is obtained through signal analysis, specifically by transforming the signal into another form. In general, each transformation transforms the input signal into a new representation in which the properties of the signal can be better detected and examined. Discrete Fourier transformation (DFT) is a form of numerical signal processing. It is used to analyze the sequence of sampled values of a certain signal changing over time and converts this time sequence into a sequence in the frequency domain according to the following relationship:

(1) $$X\left[m\right]=\overset{N-1}{\underset{n=0}{\sum }}x\left[n\right].e^{-j.2\pi .n.\frac{m}{N}}$$

where:

*X*[*m*] = M-th value of the sequence from the frequency domain (the whole sequence is also referred to as the spectrum);
*x*[*n*] = N-th sample of the time sequence;
*m* = Order of the output sample;
*n* = Order of the input sample;
*N* = Total number of input samples.

The size of the input vector *N* also determines the size of the resulting sequence in the frequency domain [31]. Looking at Equation 1, we can see that as the number of input samples *N* increases, the number of multiplication and addition operations increases. Fast Fourier transformation is an algorithm for an efficient and fast calculation of DFT. The MATLAB computational software was used to calculate the fast Fourier transformation. This software allows the FFT function to calculate the sequence of samples in the frequency domain of the input signal using the fast Fourier transformation algorithm. The calculation method is shown in the fast Fourier function:

function FastFourier(X,Fs)

L = length(X);
n = 2^nextpow2(L);
Y = fft(X,n)/L;
f = Fs/2*linspace(0,1,n/2+1);
y = 2*abs(Y(1:NFFT/2+1));
plot(f, y);

The fast Fourier function calculates and displays the sequence of samples in the frequency domain of the input signal by the fast Fourier transformation algorithm. The input parameters are the sequence of time-varying signal samples (X) and the sampling frequency of the X signal in Hz (Fs).

Robustness is a property of the method when it, by small changes of its parameters, provides the same correct and exact results. The robustness in the algorithm of vibration processing was validated by determination of the RMS (root mean square) values of the vibration signals. The RMS value of the signal represents the mean values of the signal and removes the top level and bottom level of the values, which represent the responses to the changes of the vibration system. The uncertainty of the system is included in the RMS value. The effective (RMS) value of the vibration signal *X_RMS_* is given by the equation:

(2) $$X_{RMS}=\sqrt{\frac{1}{N}\overset{N-1}{\underset{n-0}{\sum }}x{\left[n\right]}^{2}}$$

where:

*X_RMS_* = RMS value;
*x*[*n*] = *N*th sample of the time sequence;
*N* = Total number of input samples.

Autocorrelation refers to the degree of correlation of the same variables between two successive time intervals. It measures how the lagged version of the value of a variable is related to the original version of it in a time series. This procedure was performed by the Statistica software (version 10). The discrete autocorrelation *R(y)* at lag *k* for a discrete time signal *y(n)* is given by the equation:

(3) $$R_{yy}\left(k\right)=\overset{N-1}{\underset{n=0}{\sum }}y\left(n\right)\;\bar{y\left(n-k\right)}\;$$

### 2.4. Parameters Describing the Special Driving Wheel Properties

The drawbar pull measurement allows evaluation of the drawbar pull oscillation by comparison of various driving wheels. The low (load tractor in neutral) and high (load tractor in the third gear) load loads the test tractor with different driving wheel types. The test tractor was operated at the second gear and rated rotation speed of engine (3600 rpm). The test tractor velocity in the grass plot was calculated as follows:

(4) $$v=\frac{L}{t},\;\mathrm{in}\;m\;s^{-1}$$

where:

*L =* Distance of grass plot (30 m), in m;
*T—*Time, in s.

Drawbar power is a parameter suitable for evaluation of the special driving wheel properties because it includes not only drawbar pull but also vehicle speed [32,33]. Drawbar power was calculated according to the following equation:

(5) $$P_{D}=v.F_{D},\;\mathrm{in}\;W$$

where:

*v =* Tractor speed, in m s^−1^;
*F_D_* = Mean drawbar pull, in N.

The rotation speed of the test tractor driving wheels depends on actual engine speed (rated engine speed) and gear ratio (second gear) as follows:

(6) $$n_{w}=\frac{n_{e}}{i_{2}}$$

where:

*n_e_* = Actual engine speed, in s^−1^;
*i*_2_ = Gear ratio.

The FFT allows identification of the frequency that is generated by the elements (*E*) of driving wheels as follows:

- 16 elements of tire tread pattern of standard tire (rubber lugs);
- 10 elements of blade wheel;
- 4 elements of spike tire.

The test tractor uses two driving wheels on the rear driving axle. In real operation conditions, the wheels on the same axle do not have the same angular position, and in the case of special driving wheels, too. Therefore, the elements generate the frequency that belongs to the interval (*f_1_, f_2_*). The frequency *f_1_* (5) represents the minimum value and corresponds to the same angular position of the elements (blades, spikes, and rubber lugs of standard tires) of both driving wheels. The frequency *f_2_* (6) represents the maximum value and corresponds to the sum of all the elements of both wheels.

(7) $$f_{1}=E.n_{w},\;\mathrm{in}\;\mathrm{Hz}$$

(8) $$f_{2}=E.2.n_{w},\;\mathrm{in}\;\mathrm{Hz}$$

where:

*E =* Number of elements of one driving wheel;
*n_w_* = Rotation speed of driving wheels, in s^−1^.

### 2.5. Experimental Conditions

The drawbar pull measurements of the test tractor with different driving wheels were performed on the mown grass plot in the area of the Slovak Agricultural Museum in Nitra. The soil properties are listed in Table 1.

The soil properties were evaluated from three soil samples collected randomly at the depths of 0–100 mm. The sampling depth is sufficient for experiments regarding the sinkage of driving wheels during the experiments. Steel sampling cylinders with a diameter and height of 100 mm were used. To present the detailed information about the soil properties, organic matter was also evaluated because it affects the cohesion of the ground. Vegetation roots improve the shear strength properties of soils. The percentage content of vegetation roots was 0.4%. The World Reference Base for Soil Resources classifies the soil in the experimental area (southwestern region of Slovakia, Nitra district) as chernozem. Considering the particle content of sand, silt, and clay in soil samples, the soil texture is loamy sand.

## 3. Results and Discussion

### 3.1. Properties and Application of Special Driving Wheels

Both special wheels were designed to allow a change in the base and protruded position of blades or spikes. This feature of the wheels is aimed at road transportation, with the tire tread pattern of the tires but without the interactions between the blades or spikes and the ground. Releasing the safety bolt, the protruded position of blades or spikes is set by the force of wheels rotating forward. The reverse tractor motion helps place the blades or spikes to the base position. In the next development of special driving wheels, the change of the base and protruded position can be automatized and remotely controlled. Many technical solutions of special driving wheels were designed for driving wheels of agricultural machinery on rice fields, sour and peaty soil [34,35], or lunar rovers (actively actuated lugged wheels) [36] to improve the vehicle tractive properties, but they do not allow change of the lugged wheel to smooth wheels for transportation on hard landscaping.

The next most widely used measure to improve the vehicle traction performance is a change in tire inflation pressure [37,38,39]. Lower inflation pressure increases the tire contact area, improving the tire function under poor traction conditions. The ballast weight also helps improve the tractor energy efficiency. An increase in tractor load negatively affects the soil compaction, plant growth, and water transport to a great soil depth [40,41]. The blades or spikes of the special driving wheels presented in this paper improve the wheel grip without the need for decrease in tire inflation pressure or increase in tractor load.

The base condition for the correct function of both special driving wheel types is an inflation of the tires to the prescribed pressure according to the tractor manufacturer. The inflation pressure affects the deformation of the tires due to the function of the spike segments. The correct inflation ensures the correct position of the spike segments, enables the correct function of the control mechanism, and improves the tire resistance against deformation. The proposed design of the spike tires uses four spike segments. The principle of the spike tires enables using twice the number of the spike segments (eight). In this case, the forces acting on the spikes are halved to lower the tire deformation and improve the tractive performance.

The spikes have smaller dimensions in comparison with the blades. This disadvantage is partially compensated by function of the spike tires because the spikes interact with the soil compressed by the tractor weight. Spike segments negatively affect the self-cleaning ability, but the minimum number of the spike segments (four) affects the self-cleaning ability only minimally.

The blade wheels are placed near the tractor driving wheels, extending the wheel contact area. This improves the wheels’ grip. The body of the blade wheels is made of massive steel sections. The weight of the blade wheels loads the driving wheels without the need for additional ballast weight. In this case, no tire modifications are required. The disadvantage is a higher tractor width. The tractors enable changing the wheel gauge due to agrotechnical operations carried out in fields. Using the blade wheels, the wheel gauge is set so that the maximum tractor width is not exceeded.

**Figure 5:**
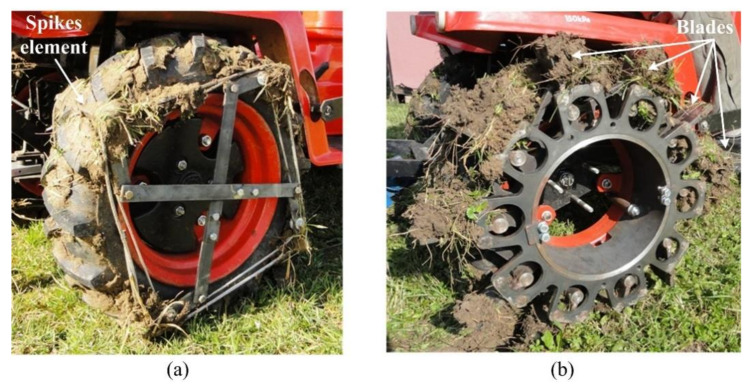
Special driving wheels covered with the soil under operation conditions on the grass plot: (**a**) spike tires; (**b**) blade wheels.

Revolving all the wheel types on the grass plot, the soil, with parts of vegetation, covered the tire tread pattern of the standard tires, the blades of the blade wheels, and the spikes of the spike tires, Figure 5. The drawbar pulls of the tractor with all the wheel types was affected by the hard surface of the grass plot, soil moisture, soil type, and the facts mentioned above.

### 3.2. Evaluation of Drawbar Pull Frequency Spectra

**Figure 6:**
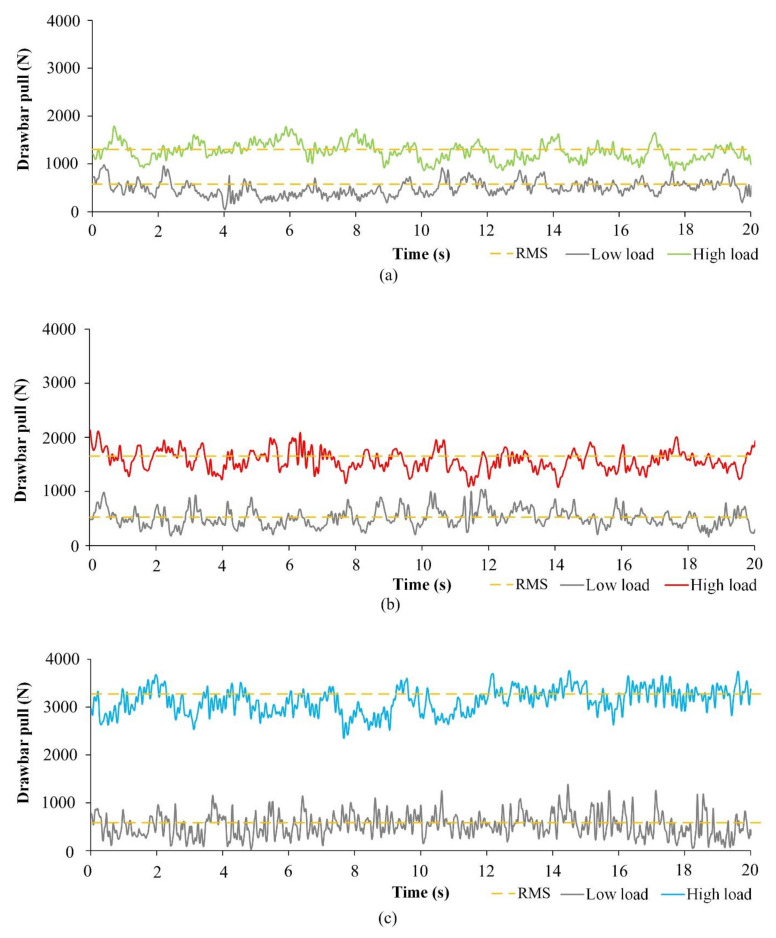
The drawbar pulls of the test tractor with low and high loads: (**a**) standard tires; (**b**) spike tires; (**c**) blade wheels.

The drawbar pulls of the test tractor with standard tires, spike tires, and blade wheels with low and high loads are shown in Figure 6. The RMS drawbar pull values of the tractor with different driving wheels with low and high loads, calculated according to Equation (2), are presented in Table 2. The levels of the drawbar RMS values are shown as straight lines in Figure 5. The RMS value of the signal represents the mean values of the signal and removes the top level and bottom level of the values, which represent the responses to the changes of the vibration system. The uncertainty of the system is included in the RMS values.

The drawbar pull oscillation depends on the type of driving wheels and affects the quality of tractor operation with special driving wheels. Hermawan et al. (1998) [42] presented that the drawbar pulls fluctuated periodically with rotation angle, and the period corresponded approximately to the interval of angular lug spacing. The designs of the special driving wheels use other lug spacing, in contrast with the tire tread pattern of standard tires. This fact, together with the shape and steel material of the blades or spikes, influenced the oscillation of drawbar pull.

The uncertainty of the drawbar pull measurements was determined.

**Figure 7:**
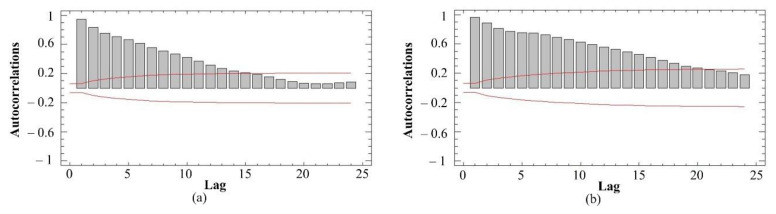

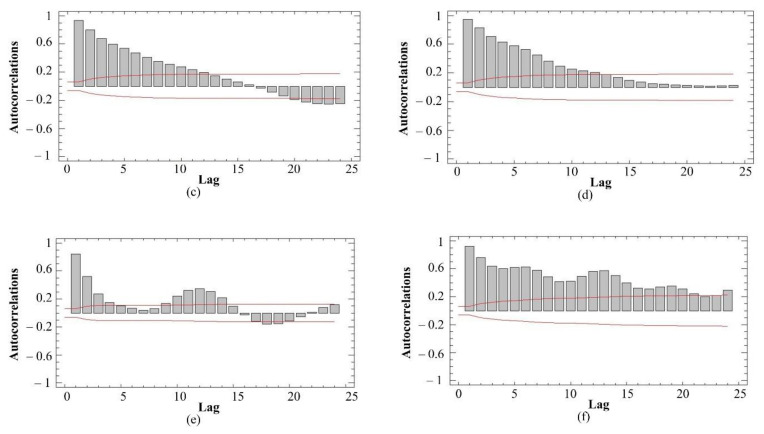
The drawbar pull signal autocorrelations: (**a**) standard tires (low load); (**b**) standard tires (high load); (**c**) spike tires (low load); (**d**) spike tires (high load); (**e**) blade wheels (low load); (**f**) blade wheels (high load).

The plots of the autocorrelation coefficients (calculated by Equation (3)) of drawbar pull signals are presented in Figure 7. It shows the estimated autocorrelations between the values of drawbar pulls at various lags. The lag autocorrelation coefficient measures the correlation between the values at time *t* and time *t-k*. The 95.0% probability limits around 0 are also shown. If the probability limits at a particular lag do not contain the estimated coefficient, there is a statistically significant correlation at that lag at the 95.0% confidence level. In the case of the standard tires, 15 of the 24 autocorrelation coefficients with a low load and 20 of the 24 autocorrelation coefficients with a high load were statistically significant at the 95.0% confidence level. In the case of the spike tires, 17 of the 24 autocorrelation coefficients with a low load and 12 of the 24 autocorrelation coefficients with a high load were statistically significant at the 95.0% confidence level. In the case of the blade wheels, 12 of the 24 autocorrelation coefficients with a low load and 22 of the 24 autocorrelation coefficients with a high load were statistically significant at the 95.0% confidence level. The results mentioned above showed that the time series may not be completely random. The drawbar pull signals were not loaded by noise with the 95.0% probability.

**Figure 8:**
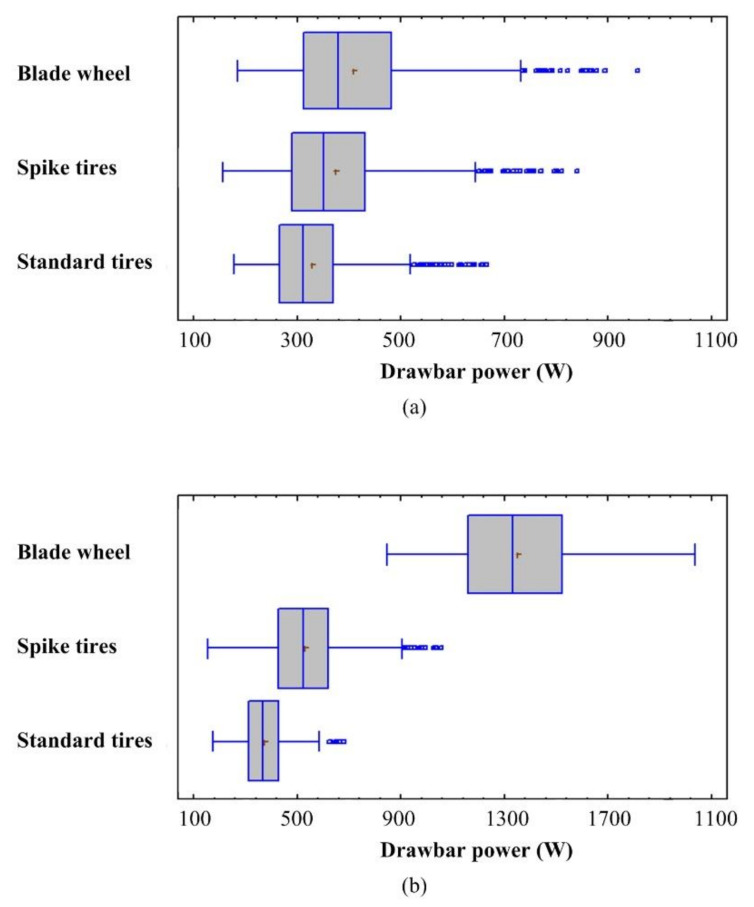
Box-and-whisker plot: (**a**) low load; (**b**) high load.

The mean drawbar pulls calculated from signals measured under experimental conditions are listed in Table 3. The special driving wheels were evaluated according to drawbar power. The one-way ANOVA determined the differences between means and the level of significance. The *p*-values test the statistical significance of each of the factors. There were statistically significant differences at the 95% confidence interval between each driving wheel type at both load levels (*p-*value < 0.05). The highest difference reached the values of 71.6% and 25.1% in the case of the blade wheels and spike tires with a high load in comparison with the standard tires. The difference reached the values of 17.2% and 12.2% in the case of the blade wheels and spike tires in comparison with the standard tires with a low load. The lower difference with a low load is caused by the tractive performance of rubber lugs in the tire tread pattern of the standard tires. The difference between various driving wheels and variation of the experimental data are shown in Figure 8. The figure also shows outliers. Their effect on the results can be considered negligible considering the sampling frequency of 1 kHz.

The rotation speed of driving wheels *n_w_* = 0.41 s^−1^ was calculated according to Equation (5) using the gear ration (*i_2_ =* 146.3) of the second gear and rated engine speed of the test tractor (*n_e_* = 3600 min^−1^). The model frequency interval was calculated according to Equations (6) and (7).

**Figure 9:**
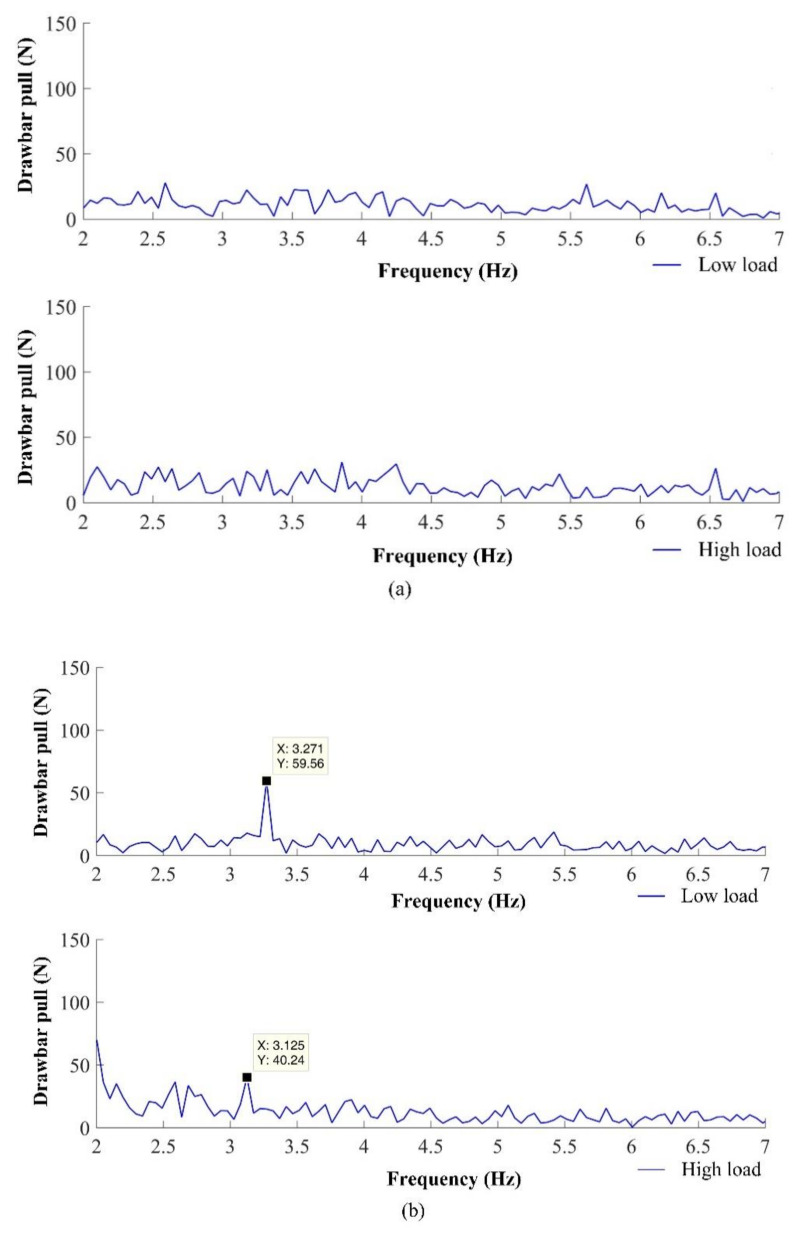

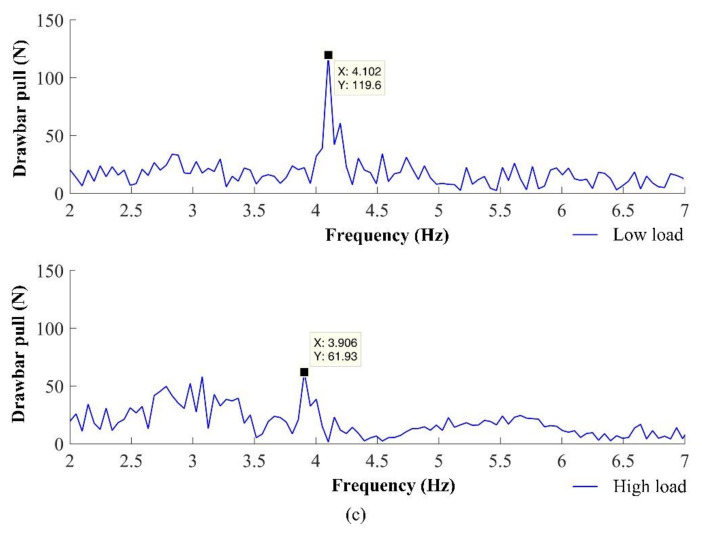
FFT of drawbar pull of the test tractor with different driving wheels with a low and high load: (**a**) standard tires; (**b**) spike tires; (**c**) blade wheels.

Figure 9 and Table 4 show that the frequency of drawbar pull oscillation cannot be identified in the case of the tire tread pattern of standard tires for two reasons. The first one is the bad yes, the change retains the meaning self-cleaning properties of the standard tires at relatively low rotation speed and at actual soil moisture. The tires were covered by the soil, and the tire tread pattern did not generate the typical frequency. The second reason can be the very low amplitude of frequency, which does not diversify in the frequency spectrum. The frequency of the tire tread pattern was not identified either in the cases of the blade wheels and spike tires.

In the spike tires and blade wheels, typical frequencies were identified and located in the calculated frequency interval.

The highest amplitudes of the frequency component resulting from spike or blade interactions with the ground were recorded at a low-load level. This fact also confirms the variation coefficient (Table 3). The sinkage of blades and spikes was only minimal with a low load. The high load affects the improvement of drawbar properties quality. The variation coefficient and amplitude of drawbar pull oscillation were lower with a high load. The lower variation coefficient is visible not only in the case of the blade wheels and spike tires but also in the case of the standard tires. Watyotha et al. (2001) [7] researched the variation in lug wheel forces depending on circumferential angle and lug spacing at three load levels. Variation was reduced by increasing the circumferential angle up to 45°. Variation was minimum at 20° lug spacing and increased as the lug spacing increased. Similarly, [43] researched the forces acting on a single model cage wheel lug. In the case of the blade wheels and spike tires, the angle of the blades or spikes was not changed because they were in the fixed position. To eliminate the undesirable vibrations, the design of both special driving wheels can be modified to set the various angles of blades or spikes. Increasing the number of lugs, [44] presented a decrease in vibrations amplitude of lugged wheels. An increase in the blade number is not possible because the blade wheels are equipped with the maximum number of blades, which is limited by the blade dimensions. The spike tires can be equipped with a higher even number of spike segments, for example six or eight. The higher number of spike segments could negatively affect the self-cleaning ability.

The spike tires generated the real frequencies 3.27 Hz with a low load and 3.13 Hz with a high load, which approximately equals the maximum frequency *f_2_* = 3.28 Hz due to 45° of angular difference in elements’ (spikes’) positions between the left and right driving wheel. On the other hand, the blade wheels generated the real frequencies 4.11 Hz and 3.91 Hz, approximately equaling the minimum frequency 4.09 Hz due to the same angular position of elements (blades) of both driving wheels.

Considering the high gear ration and rated engine speed, a decrease in the rotation speed of driving wheels due to the load is negligible and can be ignored in the case of the standard tires. The drawbar performance improvement increased the drawbar power. The difference between the drawbar power of the tractor with blade wheels and standard tires is 71.6% with a high load (Table 3). Therefore, the frequency generated by the blade wheel with a high load (3.91 Hz) was not within the model frequency interval (4.09–8.18 Hz) because of a low engine speed decrease due to high drawbar power.

The blade wheels improve the drawbar performance of the tractor in comparison with the standard tires, but they exhibit worse amplitude of drawbar pull oscillation only with a low load. With more lugs around the special driving wheels and changing the angular lugs spacing, the steadiness of forces transmission could be improved. The possibility of lugs control from the base to protruded position affects the maximum number of lugs and their shape. In the case of the blade driving wheels, a higher number of lugs requires the change of lug (blade) size regarding the lugs control.

## 4. Conclusions

The tractive performance of the special driving wheels can be evaluated according to various parameters. The analysis of drawbar pull signals based on fast Fourier transformation was applied to evaluate the operation of the blades wheels, spike tires, and standard tires. The results showed that the interactions between the blades or spikes and the ground were identified in frequency spectra. The highest amplitude of the frequency component was observed in the case of the blade wheels and corresponds with the highest variation of measured drawbar pull signals. The lower amplitude was detected in the spike tires due to the lower dimensions of spikes in comparison with blades. In the case of the standard tires, the frequency component typical for the rubber lugs of the tire tread pattern was not identified in the frequency spectrum. The high number of rubber lugs and that the tire tread pattern became covered with the soil were the main reasons. The absolute values of amplitudes were higher in the case of low load in comparison with high load. The highest difference of drawbar pulls was reached in the blade wheels in comparison with the standard tires. In the case of the spike tires, the difference reached lower values due to the lower dimensions of spikes in comparison with blades. Besides the drawbar pull increase, the tractor velocity also increased due to the special driving wheels. Evaluating the driving wheels according to the drawbar power, the difference between both special driving wheels and the standard tires was expressed more complexly. The special driving wheels reached a statistically significant difference in comparison with the standard tires. The results showed that the analysis of the frequency spectrum of the drawbar pull signals identified the frequency components generated by the spikes or blades. The frequency spectrum analysis showed important characteristics in the operation of the special driving wheels.

The results identified the frequency components resulting in the vibrations due to interaction between the spikes or blades and the ground. Vibrations negatively affect the tractor operation and there are some solutions to eliminate them. Therefore, future work will be focused on the application and testing of the special driving wheels innovated to improve the tractive performance and eliminate the vibration level. The main innovation will be a sharpening of the spikes and blades to improve the penetration to the ground. In the case of the spike tires, it is possible to increase the number of spike segments to eight around the wheel circumference. It enables more spikes to be in contact with the ground at the same time. In the case of the blade wheels, it is not possible to increase the blade number, but it is possible to decrease the length of the blades. The shorter blades decrease the lifting forces. Further research will also focus on automation and remote control of both types of special wheels.

## Figures and Tables

**Table 1:** Soil properties.

Parameter	Unit	Value
gravel	%	15.3
sand	%	45.2
silt	%	35.4
clay	%	4.1
organic matter	%	6.62
particle density	g m^−3^	2.6
moisture content (dry basis)	%	27.7

**Table 2:** Drawbar RMS values of the tractor with different driving wheels with low and high loads.

Wheel Type	Load Level	RMS Drawbar Pull, in N
standard tires	low	518.056
	high	1274.652
spike tires	low	539.295
	high	1589.953
blade wheels	low	580.914
	high	3141.954

**Table 3:** Drawbar parameters of the tractor with different driving wheels with low and high loads.

Wheel Type	Load Level	Tractor Speed, in m s^−1^	Mean Drawbar Pull, in N	Standard Deviation, in N	Variation Coefficient, in %	Drawbar Power, in W
standard tires	low	0.667	493.9	155.3	31.5	329.5
	high	0.306	1260.4	185.4	14.7	385.7
spike tires	low	0.731	513.4	164.1	31.9	375.4
	high	0.326	1578.3	184.9	11.7	514.5
blade wheels	low	0.751	530.5	236.1	49.6	398.4
	high	0.434	3129.4	261.4	8.4	1358.2

**Table 4:** Frequencies corresponding to the elements (tire tread pattern, blades, and spikes) of different driving wheels.

Wheel Type	Load	Rotation Speed of Driving Wheels, in s^−1^	Model Frequency Interval		Experimental Frequency, in Hz
			Minimum Frequency, in Hz	Maximum Frequency, in Hz	
standard tires	low	0.41	6.56	13.12	-
	high				-
spike tires	low		1.64	3.28	3.27
	high				3.13
blade wheels	low		4.09	8.18	4.11
	high				3.91

## Data Availability

Not applicable.
